# Pathological Crosstalk between Metastatic Breast Cancer Cells and the Bone Microenvironment

**DOI:** 10.3390/biom10020337

**Published:** 2020-02-19

**Authors:** Jennifer Zarrer, Marie-Therese Haider, Daniel J. Smit, Hanna Taipaleenmäki

**Affiliations:** 1 Molecular Skeletal Biology Laboratory, Department of Trauma, Hand and Reconstructive Surgery, University Medical Center Hamburg-Eppendorf, 20246 Hamburg, Germany; 2 Institute of Biochemistry and Signal Transduction, University Medical Center Hamburg-Eppendorf, 20246 Hamburg, Germany

**Keywords:** breast cancer, bone metastases, bone microenvironment, osteoclast, osteoblast, osteocyte, adipocyte, vasculature, hypoxia, nerve cells

## Abstract

Bone is the most common metastatic site in breast cancer. Upon arrival to the bone, disseminated tumor cells can undergo a period of dormancy but often eventually grow and hijack the bone microenvironment. The bone marrow microenvironment consists of multiple cell types including the bone cells, adipocytes, endothelial cells, and nerve cells that all have crucial functions in the maintenance of bone homeostasis. Tumor cells severely disturb the tightly controlled cellular and molecular interactions in the bone marrow fueling their own survival and growth. While the role of bone resorbing osteoclasts in breast cancer bone metastases is well established, the function of other bone cells, as well as adipocytes, endothelial cells, and nerve cells is less understood. In this review, we discuss the composition of the physiological bone microenvironment and how the presence of tumor cells influences the microenvironment, creating a pathological crosstalk between the cells. A better understanding of the cellular and molecular events that occur in the metastatic bone microenvironment could facilitate the identification of novel cellular targets to treat this devastating disease.

## 1. Introduction

The development of bone metastasis is a selective, multistep process. It involves colonization of the bone marrow, survival and/or dormancy of disseminated tumor cells (DTCs) in supportive microenvironments (niches), and ultimately reactivation and progression into actively proliferating metastases [1]. Due to the various challenges faced upon arrival at the secondary site, this process has been described to be less efficient than tumor growth at the primary site. Eventually, only a small subset of DTCs initiate metastatic growth [1,2]. In addition to lung, brain, and liver, breast cancer metastases are primarily found in bone, a phenomenon already observed by Steven Paget [3]. He linked the ”seed and soil” theory that ”when a plant goes to seed, its seeds are carried in all directions; but they can only live and grow if they fall on congenial soil” to the establishment of breast cancer bone metastasis [3]. Bone offers an ideal soil for DTCs (seeds) as it continuously undergoes remodeling, and therefore supplies a variety of soluble factors including transforming growth factor β (TGF-β), interleukins (ILs) and calcium (Ca^2+^) that foster tumor growth. In addition, the bone is highly vascularized tissue. Despite the dense vascular network, bone marrow is also considered to be a tissue with limited oxygen supply, and therefore the bone marrow microenvironment is a hypoxic environment. Here, we review the recent literature on the cellular composition and properties of the physiological bone microenvironment and discuss how the complex microenvironment regulates the process of cancer-induced bone disease with a focus on breast cancer bone metastases.

## 2. The Cellular Composition of the Bone Microenvironment

Hematopoietic and mesenchymal stem cells (HSCs and MSCs, respectively) give rise to the various cell populations found in the bone microenvironment. Cells of hematopoietic origin include lymphoid and myeloid linage cells that finally differentiate into immune cells, megakaryocytes, myeloid cells (monocytes/macrophages/granulocytes), and erythrocytes [4]. Adipocytes, bone, and connective tissue forming cells such as chondrocytes, osteoblasts, and myoblasts arise from MSCs. Multinucleated osteoclasts arise from the monocyte-macrophage lineage and are responsible for resorbing the bone matrix. Cytokines that drive their differentiation into active, bone resorbing osteoclasts include macrophage-colony stimulating factor (M-CSF) and receptor activator of nuclear factor kappa-β ligand (RANKL), both mainly produced by osteoblasts in the bone [5].

Osteoblasts are derived from MSCs and are responsible for forming new bone matrix. Osteoblast differentiation is regulated by several signaling pathways and transcription factors including parathyroid hormone (PTH), wingless (Wnt), runt-related transcription factor 2 (Runx2) and osterix (Osx) [6]. Mature bone-forming osteoblasts are characterized by the secretion of bone matrix proteins including collagen I (Col1), alkaline phosphatase (ALP), and osteocalcin. After a short phase of active bone formation, osteoblasts are embedded in the bone, upon which they are classified as osteocytes, or adapt a quiescent state on the bone surface as a so-called bone lining cell [7].

Located within the bone matrix, osteocytes are the most abundant cell type in bone comprising about 90% to 95% of all bone cells in the adult skeleton. Initially, osteocytes were described as passive cells, however, their role in transducing mechanical forces and controlling both bone formation and resorption is now well established [8]. By secreting the Wnt-signaling antagonists sclerostin and dickkopf-related protein 1 (Dkk1), osteocytes negatively regulate osteoblast function and viability. In addition, osteocytes are thought to be a major source of RANKL in the bone, therefore, controlling osteoclast differentiation and function [9,10].

Similar to osteoblasts, bone marrow adipocytes arise from MSCs through a tightly controlled differentiation process. One of the key transcription factors that promote adipogenesis is peroxidase proliferator-activated receptor γ (PPARγ). Increased activation of PPARγ directs MSCs into adipogenic lineage while reduction or inhibition of PPARγ in MSCs leads to decreased adipogenesis and simultaneously increased osteogenesis [10]. Conversely, Wnt signaling-mediated activation of β-catenin not only stimulates osteogenesis, but also inhibits adipogenesis. In addition to cell intrinsic mechanisms, adipocytes affect bone cells by secreting cytokines and signaling proteins, including tumor necrosis factor alpha (TNF-α). Secreted adipokines have been shown to activate the nuclear factor kappa beta (NF-kB) signaling in osteoblasts suppressing their differentiation [11]. Interestingly, bone marrow adipocytes also regulate osteoclast differentiation. TNF-α treatment induces RANKL expression and secretion in bone marrow adipocytes which stimulates osteoclast differentiation and activation [12] emphasizing the role of bone marrow adipocytes in regulating skeletal homeostasis.

In addition, a dense, interconnected vascular system comprises the bone marrow, thereby maintaining hematopoiesis and osteogenesis [13]. The inner layer of the blood vessels is composed of endothelial cells, which are covered by pericytes or vascular smooth muscle cells [14]. Different subtypes of endothelial networks have been identified in the bone, type L and type H endothelium, which can be distinguished based on their expression markers and morphological properties [13]. The type H capillaries are localized underneath the growth plate in the metaphysis and in the endosteum and have a high expression of CD31 (PECAM1) and endomucin (CD31^hi^Emcn^hi^). Osterix positive osteoprogenitor cells are located in close proximity to type H endothelium [13]. The type L capillaries, connected through a transition vessel type to type H endothelium, are found in the diaphysis of the long bones and have a low expression of CD31 and endomucin (CD31^lo^Emcn^lo^). They form the sinusoidal vasculature of the long bone [15], draining into the central vein which is associated with the presence of hematopoietic cells [13]. Despite the high abundance of different and highly connected blood vessels in the bone, the oxygen tension levels fluctuate throughout the bone marrow compartments, as well as in the endosteum and periosteum. Only arteries supply the bone with arterial oxygen via the type H vessels to the metaphysis of the bone. There is only low oxygen supply in the diaphysis of the long bones, and thus the bone marrow is a hypoxic environment [16].

Although nerve cells are often less recognized in the context of bone microenvironment, the bone marrow is innervated with sympathetic, sensory, and glutaminergic nerves [17]. Sympathetic nerves are found in the cortex and release neurotransmitters, including norepinephrine, into the bone marrow. Binding of norepinephrine to the β2-adrenergic receptor (β2AR), expressed on osteoblasts, leads to an upregulation of RANKL and consequently increased osteoclastogenesis and bone resorption [18,19]. Therefore, the nervous system is also an important mediator of bone modeling and bone remodeling.

## 3. Physiological and Pathological Bone Remodeling

Bone is a dynamic tissue that is constantly remodeled throughout life. Under physiological conditions, bone is continuously degraded by osteoclasts and new bone matrix is produced by osteoblasts [20]. These highly balanced and tightly regulated actions maintain bone mass constant in healthy individuals [21]. Bone remodeling requires coordinated molecular communication between osteoclasts and osteoblasts. For instance, osteoblasts and osteocytes provide differentiation signals to osteoclasts through membrane-bound or soluble forms of RANKL. RANKL binds to the RANK receptor on osteoclasts promoting their maturation and function. Osteoblasts can also negatively regulate osteoclast differentiation by secreting osteoprotegerin (OPG), a soluble decoy receptor for RANKL. Conversely, osteoclasts influence osteoblast differentiation and activity by secreting semaphorin 4D (Sema 4D) and other factors [22]. In addition, several cytokines released from the bone matrix, including TGF-β and insulin-like growth factor 1 (IGF-1) regulate osteoblast and osteoclast function. Furthermore, secreted matrix metalloproteinases (MMPs) have an important role in the skeleton during endochondral ossification, and regulate MSC differentiation, recruitment of osteoclasts, and bone remodeling [23].

**Figure 1:**
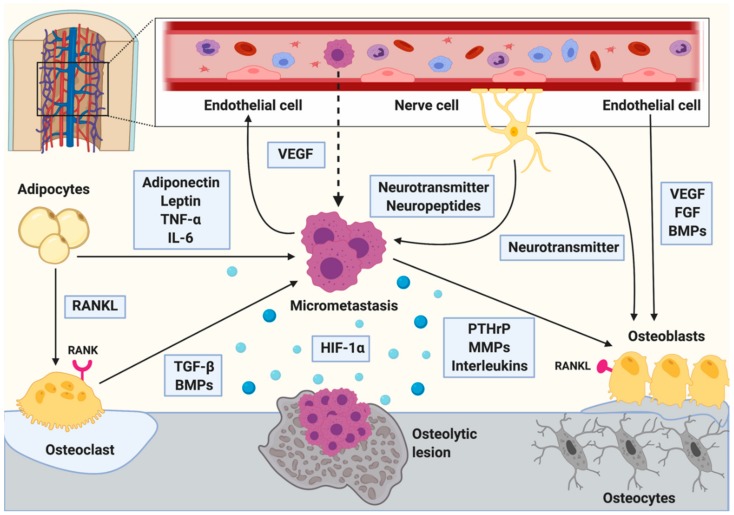
Schematic illustration of the metastatic bone microenvironment. The bone marrow contains various cell types including skeletal cells, nerve cells, endothelial cells, and adipocytes. Tumor cell invasion disrupts physiological bone homeostasis leading to an abnormal expression of many growth factors, cytokines, chemokines, and signaling pathways. Osteoblasts are responsible for new bone formation and express receptor activator of nuclear factor kappa-β ligand (RANKL) which binds to the RANK-receptor on osteoclasts resulting in their maturation. Mature osteoclasts resorb the bone matrix which releases growth factors, including transforming growth factor β (TGF-β) and bone morphogenic proteins (BMPs) into the bone marrow. TGF-β not only stimulates osteoblast precursor cells, but also the tumor cells. Adipocytes promote osteoclastogenesis by expressing RANKL. In addition, they affect tumor cell proliferation by the expression of leptin, adiponectin, tumor necrosis factor α (TNF-α), and interleukin 6 (IL-6). The perivascular niche is critical for the interaction of tumor cells with the bone. Tumor cells are often located close to the endothelial cells and secrete vascular endothelial growth factor (VEGF) to induce angiogenesis. Endothelial cells also support osteoblast differentiation through the expression of VEGF, fibroblast growth factor (FGF) and BMPs. Nerve cells are wrapped around the vessels and provide neurotransmitters to osteoblasts, as well as neurotransmitters and neuropeptides to the tumor cells. In addition, hypoxia increases tumor growth and invasiveness through hypoxia-inducible factor-1α (HIF-1α) signaling. The tumor cells support their own growth by secreting VEGF, parathyroid hormone related protein (PTHrP), matrix metalloproteinases (MMPs), and interleukins that promote RANKL expression in osteoblasts. The Figure was created with Biorender (Biorender.com).

Any imbalance in bone remodeling affects bone mass, ultimately leading to pathological conditions. Especially during aging, but also in various disease conditions (e.g., kidney or liver disease) or in response to the treatment with certain drugs such as glucocorticoids, bone resorption often increases, and bone formation decreases. This leads to a reduction of bone mass and bone mineral density, frequently resulting in osteoporosis and related fractures [20]. In addition, several cancers including breast and prostate cancer, multiple myeloma, and renal cell carcinoma can alter bone remodeling and bone homeostasis. Once tumor cells have colonized the bone, they hijack stromal cells (i.e., osteoblasts and osteoclasts) for their own benefit (Figure 1). Therefore, the finely tuned balance of bone resorption and formation is destroyed resulting in osteolytic, osteoblastic, or mixed bone lesions. In breast cancer, the lesions are in most cases osteolytic [24].

## 4. Bone Cells Regulating Breast Cancer Bone Metastasis

### 4.1. The Role of Osteoclasts in Breast Cancer Bone Metastasis

Osteolytic disease in breast cancer bone metastasis proceeds upon tumor cell-induced upregulation of osteoclast activity. Hence, osteoclasts are well established as key drivers of the disease [25]. Several tumor-derived factors (e.g., ILs) have been reported to stimulate osteoclast differentiation, maturation, and activity (reviewed in greater detail in [26,27,28]). For example, conditioned medium from MDA-MB-231 breast cancer cells has been shown to promote osteoclastogenesis of mouse bone marrow cells in vitro. The development and survival of osteoclast progenitors were reduced after using an IL-11 neutralizing antibody suggesting that breast cancer cells can promote osteoclastogenesis, at least partially, through IL-11 [29]. Whereas these data show that tumor cells directly stimulate osteoclasts, they can also indirectly induce osteoclastogenesis and bone destruction by altering the RANKL/OPG production of osteoblasts and osteocytes [30]. Parathyroid hormone-related protein (PTHrP), for example, is well known to increase RANKL expression in osteoblasts, and therefore supports osteoclast formation [30]. Altered serum RANKL/OPG ratios are also especially prominent in patients with severe osteolysis as compared with healthy individuals [31]. In turn, bone-derived factors that are released during the excessive, osteoclast-mediated bone resorption (Ca^2+^, TGF-β, and IGFs) further stimulate tumor growth. Thereby, the so-called “vicious cycle” between bone destruction and tumor growth is fueled [32].

While osteoclasts drive the late stage of breast cancer bone metastases, they are suggested to be less important during the early stages of bone metastases. This is supported by studies from Wang et al. that recently characterized the cellular composition of the stromal cells surrounding both triple negative and estrogen-receptor positive breast cancer micrometastases in vivo (MDA-MB-231 and MCF-7, respectively) [33]. The presence of tartrate-resistant acid phosphatase (TRAP) positive osteoclasts was increased during the transition from nonproliferative to overt osteolytic metastases, whereas the percentage of osteoclastic cells surrounding breast cancer micrometastasis was below 20%. Furthermore, cathepsin K-positive osteoclasts were not found to be in direct contact with the cancer cells [33]. In addition, others have shown that inhibition of osteoclasts with the bisphosphonate zoledronic acid did not inhibit or reduce breast cancer cell homing to bone in vivo [34], suggesting that tumor cell homing to bone is probably not regulated by osteoclasts.

### 4.2. The Role of Osteoblasts in Breast Cancer Bone Metastasis

As discussed in the previous section, our current knowledge on how osteoclasts drive the progression of breast cancer bone metastases is well established [32]. In contrast, the contribution of osteoblasts to disease establishment has been poorly investigated. Recently, research has moved away from the concept that osteoclasts alone drive the progression of breast cancer bone metastasis, and osteoblasts have been increasingly investigated as novel cellular targets [33,35]. In fact, the contribution of osteoblasts to the establishment and progression of bone metastasis might be underappreciated in the concept of the vicious cycle. Tumor-derived factors including PTHrP are known to stimulate RANKL expression on osteoblasts, resulting in an increased osteoclast activity [32]. By this mechanism, osteoblasts have somewhat been considered as indirect regulators of osteolytic disease. In addition, it is well known that osteoblast numbers decline during disease progression. However, recent studies suggest a key role of osteoblasts during the earliest stages of bone metastasis such as tumor cell homing to bone and dormancy [33,35,36], which are stages of the disease that still remain challenging to investigate.

#### 4.2.1. Osteoblasts during the Early Stages of Bone Metastasis

It has been proposed that the dissemination of tumor cells to distant organs could be regulated similarly to HSC homing and retention. One key regulatory pathway of the HSC niche includes the C-X-C motif chemokine ligand 12 (CXCL12)/C-X-C chemokine receptor type 4 (CXCR4) signaling axis, with osteoblasts as potential key players [37,38]. CXCL12 (also known as stromal cell-derived factor 1 (SDF-1)) is expressed by a variety of cells of the bone microenvironment including osteoblasts and endothelial cells [39] while cancer cells including breast cancer cells express CXCR4 [40], the receptor for CXCL12. Findings by Price et al. showed that the CXCL12–CXCR4 interaction between breast cancer cells and stromal cells is important for anchoring disseminated breast cancer cells in the bone marrow [41]. Interestingly, inhibition of this signaling axis resulted in mobilization of dormant tumor cells into the circulation. Consistently, disseminated breast cancer cells are frequently localized close to trabecular bone areas below the growth plate cartilage that is enriched in osteoprogenitor cells (osteopontin (OPN)^high^ and CXCL12^high^) [42]. Similarly, MDA-MB-231 breast cancer cells have been shown to preferentially colonize close to trabecular bone areas in the metaphysis of BALB/c nude mice, areas of the bone that are usually rich in osteoblasts [13,34,43]. These findings support a key role of osteoblasts during tumor cell homing and colonization of the bone marrow.

Evidence from in vitro studies investigating the interaction between osteoblasts and breast cancer cells supports this hypothesis. By using the transwell migration assay Bussard et al. showed that MDA-MB-231 breast cancer cell migration was increased when they were allowed to migrate towards medium conditioned by osteoblasts [44]. Similar results have been reported by Vallet et al. using the wound healing migration assay. ALP^low^, OPN^low^, Runx2^high^, Osx^high^, and CD166^high^ preosteoblasts, but interestingly not mature osteoblasts, increased the migration of MDA-MB-231 breast cancer cells [45]. By showing that the adhesion of breast cancer cells to preosteoblastic cells was strongly increased as compared with undifferentiated or mature osteoblasts, the authors further strengthen the proposed role of osteoblasts as key regulators during the early stages of bone metastasis [45]. In contrast, a specific subtype of osteoblasts has been shown to retard breast cancer cell proliferation [35]. Conversely, other studies have also shown that breast cancer cells can modify osteoblast behavior including their migration [46]. Using an admix model by intrafemoral co-injection of breast cancer cells (MDA-MB-231 and MDA-MB-435) and osteoblasts (MC3T3-E1) Bodenstine et al. showed that co-injected mice developed larger tumors as compared with mice that were injected with tumor cells alone [47]. Therefore, the authors strengthen the hypothesis that osteoblasts and breast cancer cells communicate to promote tumor growth.

Once in the secondary organ, tumor cells can remain dormant until a yet unknown signal causes them to initiate metastatic growth. Although it still remains unknown which signals trigger the escape of tumor cells from dormancy and causes them to transition into actively proliferating metastases, it has been proposed that osteoblasts could be involved in this process. It is hypothesized that the presence of tumor cells causes osteoblasts to produce soluble factors that could not only act as chemo-attractants but also as maintenance or growth factors for DTCs [48]. The production of inflammatory cytokines such as IL-6, IL-8, and monocyte chemoattractant protein-1 (MCP-1) in both, human hFOB 1.19 and murine M3T3-E1 osteoblasts was increased upon the interaction with metastatic MDA-MB-231 breast cancer cells [48]. Similarly, an increase in osteoblast derived cytokines (e.g., IL-6, IL-8, MCP-1, macrophage-inflammatory protein 2 (MIP-2), and vascular endothelial growth factor (VEGF)) has been identified upon the presence of MDA-MB-231 breast cancer cells. This was demonstrated both, in vitro using conditioned medium, as well as in ex vivo cultures of tumor bearing bones from athymic mice [44]. Importantly, these inflammatory cytokines are well known to be involved in regulating breast cancer cell invasiveness and metastasis [49,50].

Furthermore, it has been proposed that the microenvironment of breast cancer micrometastasis is primarily composed of osteoblastic cells [33]. Using in vivo mouse models and both triple negative MDA-MB-231 and estrogen-receptor positive MCF-7 cells, the authors showed an increase of TRAP-positive osteoclasts once the micrometastases were transitioning into actively proliferating metastases. In contrast, the tumor stroma surrounding the indolent, nonproliferative micrometastases prior to the osteolytic stage, was predominantly of osteoblastic origin. Indeed, the majority of the cells adjacent to the breast cancer micrometastases abundantly expressed markers of the osteoblastic lineage (ALP, Col1) [33]. The presence of osteoblastic cells was also increased in tumor bearing bones as compared with tumor-free bones, suggesting that osteoblasts facilitate breast cancer growth in bone. The authors propose that the osteoblast–breast cancer cell interaction might be regulated via E- and N-cadherins, consequently resulting in an enhancement of mammalian target of rapamycin (mTOR) activity in the cancer cells. The increase in mTOR signaling in cancer cells suggests a potential mechanism by which osteoblasts would regulate breast cancer cell dormancy in the bone [33].

While the majority of studies suggest a tumor promoting function of osteoblasts and that they would rather initiate metastatic growth, Kolb et al. reported the existence of an osteoblast subtype that could actually suppress breast cancer growth [35]. This subpopulation of osteoblasts was educated upon contact with tumor cells in an OPN^high^ and alpha smooth muscle actin (aSMA)^low^ phenotype in vivo. Recapitulating this osteoblast phenotype in vitro by using MC3T3-E1 osteoblasts and conditioned medium from MDA-MB-231 and MCF-7 breast cancer cells, the authors showed that this subpopulation expressed lower levels of the inflammatory cytokine IL-6 but increased levels of extracellular matrix remodeling proteins including Col1 and MMP3. Importantly, a conditioned medium from these osteoblasts reduced breast cancer cell proliferation in vitro, suggesting a novel way how breast cancer cells, aided by osteoblasts, might enter dormancy [35].

#### 4.2.2. Osteoblasts during Later Stages of Bone Metastases

Although the role of osteoblasts during the earlier stages of bone metastases is increasingly appreciated and investigated, their contribution to advanced bone metastases remains unknown. This could result from the fact that osteoblast numbers decline during disease progression [36,51]. This has already been proposed by Phadke et al., in 2006, who showed that breast cancer micrometastases were located in great proximity to osteoblastic cells and that the number of osteoblasts was reduced as tumor burden increased [51]. Consistently, studies by Brown et al. reported an increase in osteoblast numbers prior to the onset of osteolytic disease, followed by a reduction in the osteoblast/osteoclast ratio once osteolytic lesions were predominant [36]. Breast cancer cells or their conditioned medium can also induce osteoblast apoptosis, and inhibit osteoblast differentiation and adhesion [52,53]. Hence, not only by increasing osteoclast activity but also by inducing osteoblast apoptosis, tumor cells stimulate bone destruction during the late stages of bone metastasis.

#### 4.2.3. The Effect of Cancer Cell–Osteoblast Interactions on Bone Matrix and Bone Quality

Studies have suggested that the interaction between metastatic cancer cells and bone cells including osteoblasts and osteocytes does not only contribute to tumor growth but could also influence bone matrix organization, as well as bone quality. It is well established that the composition and structure of the bone matrix are important for bone mechanical stability. In a physiological setting, bone matrix is characterized by an anisotropic aligned structure of collagen fibers and c-axis orientated apatite crystals, which is important to maintain the mechanical properties of the bone. Alterations of this alignment are associated with various diseases and disrupted mechanical function. For instance, metastases-bearing bones have disorganized collagen and apatite alignment and disturbed microstructure, as demonstrated by Sekita and colleagues [54]. Using in vivo models and various imaging techniques, the authors showed that osteoblastic prostate cancer cells (MDA-PCa-2b) [54,55], as well as osteolytic breast cancer (MDA-MB-231) and melanoma (B16F10) cells [55], disrupt the anisotropic collagen/apatite structure and the alignment of osteocytes, resulting in an inappropriate mechanical function of metastases-bearing bone. The underlying cellular mechanism was shown to be an abnormal arrangement of osteoblasts upon physical contact with cancer cells [55]. This direct cell-cell contact resulted in heterogenous osteoblast positioning, altered osteoblast shape, and disrupted intercellular junctions. In addition, direct contact with MDA-MB-231 breast cancer cells interfered with osteoblast cell division [56]. Another study using an athymic rat model with mixed osteoblastic and osteolytic bone metastasis showed that metastatic lesions had an impact on bone structural properties with no effect on mechanical characteristics of the vertebrae possibly due to the osteoblastic lesions [57].

### 4.3. The Role of Osteocytes in Breast Cancer Bone Metastasis

Although osteocytes were originally not included in the concept of the vicious cycle of bone metastasis, several aspects support their role in breast cancer bone metastasis. Whether this is due to a direct interaction with disseminated tumor cells or through indirect actions on bone cells including endothelial cells, osteoblasts, and osteoclasts remains to be elucidated [58].

So far, studies investigating the role of osteocytes in bone metastasis have primarily been performed in models of multiple myeloma [59,60,61] and knowledge on breast cancer remains limited. However, osteocytes secrete various growth factors and cytokines known to have key roles in breast cancer cell migration, proliferation, and the vicious cycle (e.g., RANKL, MMPs, TNFα, sclerostin [24,62]). Hence osteocytes could also directly affect disseminated breast cancer cells in bone. In vitro studies using human metastatic breast and prostate cancer cells support this hypothesis [63]. Conditioned medium from MLO-Y4 osteocytes increased the proliferation of human breast (MDA-MB-231 and MCF-7) and prostate cancer cells (DU145 and PC3). The authors also reported a reduced wound healing capacity of MDA-MB-231 cells upon stimulation with osteocyte conditioned medium in a scratch-wound assay, whereas no effect was observed using MCF-7 cells. Interestingly, osteocyte conditioned medium increased the transwell migration but not the transwell invasion capacity of MDA-MB-231 breast cancer cells [63].

Osteocytes could also be involved in regulating the early stages of breast cancer bone metastasis including the homing and colonization to bone. Interestingly, conditioned medium from mechanically stimulated MLO-Y4 osteocyte-like cells was able to reduce the adhesion of metastatic MDA-MB-231 breast cancer cells to human umbilical vein endothelial cells (HUVECs) [58]. In addition, osteocyte conditioned medium reduced endothelial cell permeability, as well as breast cancer cell invasion in vitro. Furthermore, the crosstalk between mechanically stimulated osteocytes and endothelial cells decreased the expression of MMP-9, an enzyme involved in metastatic cancer cell migration, in breast cancer cells [58]. These findings not only strengthen the importance of mechanical loading (i.e., exercise) in preventing bone metastases, but also highlight a key role of osteocytes in the establishment of metastatic breast cancer. Another aspect that supports a regulatory role of osteocytes during early stages of breast cancer bone metastasis is that osteocytes produce CXCL12 [64], a chemokine involved in tumor cell homing and dormancy [65,66]. Through activation of the CXCL12-CXCR4 signaling cascade in cancer cells, osteocytes could consequently mediate cancer cell homing to bone. Importantly, there has been no data that directly links osteocytes to tumor cell dormancy. However, given their critical role in bone remodeling it might be possible that osteocytes indirectly regulate tumor cell dormancy via their action on osteoblasts and/or osteoclasts.

## 5. Adipocytes as Regulators of Bone Metastases

Bone marrow adipocytes store and secrete lipids, adipokines, and cytokines, thereby influencing the cells in the bone marrow through autocrine, endocrine, and paracrine signaling [67]. Bone marrow adipose tissue increases during aging. In children, approximately 15% of bone volume consist of adipocytes, whereas adolescent long bone marrow is composed of 70% of bone marrow adipocytes [68,69]. For a long time, bone marrow adipocytes were considered as “filler” cells of the bone marrow. However, recent studies have demonstrated that bone marrow adipocytes act as an endocrine organ. For example, bone marrow adipocytes alter the metabolism of tumor cells, stimulate tumor cell adhesion, colonization, and proliferation, and promote resistance to chemotherapy [69]. Consistently, obesity and high fat diet have been associated with increased tumor growth and bone destruction [67,70,71].

Adipocytes affect cancer cells through various mechanisms. Adipocytes contain lipid droplets which are a source of fatty acids. Interestingly, adipocytes located close to tumor cells have been shown to transport their lipid droplets to the cancer cells, and therefore serve the cancer cells as a source of energy and impacting their metabolism [67]. Lipids arising from adipocytes have been demonstrated to increase tumor growth and invasiveness by increasing the expression of fatty acid binding protein 4 (FABP4), a fatty acid chaperone that is involved in glucose and lipid metabolism, heme oxygenase 1 (HMOX), and IL-1β in metastatic tumor cells. The loss of lipids in adipocytes was also demonstrated in vitro upon co-culture with 4T1 breast cancer cells leading to a higher invasiveness of cancer cells [72]. In addition, adipocytes can influence tumor cells by secreting inflammatory cytokines and growth factors, chemokines, and adipokines. Adipocyte-secreted factors, such as IL-6, TNF-α, and CXCL12 support tumor development and proliferation, as well as inhibit apoptosis in multiple myeloma [73,74]. This tumor promoting function was also observed in prostate cancer in which the secretion of CXCL1 and CXCL2 enhanced osteoclastogenesis and resulted in an increased cancer cell survival [68].

Leptin and adiponectin, the most important adipokines, have partially contradictory functions in cancer cell behavior. Adiponectin is involved in glucose uptake and in fatty acid breakdown. Adiponectin has an antitumoral effect in the skeleton by stimulating apoptosis and decreasing proliferation of breast cancer cells. This is achieved by the activation of different pathways including mTOR and NF-_K_B signaling in the breast cancer cells. Unfortunately, this antitumor effect is diminished in obesity due to reduced expression of adiponectin receptors leading to therapy resistance [75]. Controversially, other studies have shown that adiponectin can stimulate breast cancer migration and growth [76,77]. In contrast, leptin has a protumor effect and is associated with increased metastasis [75,78]. For example, adipocyte-derived leptin and IL-1β increase breast cancer cell colonization and migration to the adipose tissue adipocytes of the skeleton [79]. Interestingly, leptin is also involved in the regulation of VEGF in breast cancer cells via hypoxia-inducible factor 1α (HIF-1α) and NF-κB signaling promoting cancer progression [80].

As discussed above, adipocytes have emerged as interesting mediators of metastatic bone disease. However, more research should be performed to better understand the molecular and cellular interactions between adipocytes and metastatic breast cancer cells in the bone microenvironment.

## 6. Bone Marrow Vasculature and Hypoxia as Regulators of Metastatic Bone Disease

### 6.1. The Role of Bone Marrow Vasculature in Bone Metastases

The bone marrow is a highly vascularized tissue. The blood vessels not only provide the bone with oxygen and nutrients, but also remove metabolites and transport different types of hormones, neurotransmitters, growth factors and cells, including cancer cells [13,14]. The endothelial cells in the inner layer of the blood vessels form the first semipermeable barrier for the circulating tumor cells to reach the bone marrow.

A rearrangement of the vascular network has been associated with various diseases including osteoporosis, osteosclerosis, and diabetes mellitus [81,82]. Similarly, the structured organization of blood vessels change to a disorganized network of tortuous vessels in the presence of breast cancer cells in the bone, suggesting that the vasculature could have a role in the development and progression of metastatic bone disease [83]. Indeed, bone metastatic breast cancer cells preferentially localize in the metaphysis of the long bones adjacent to the endothelial cells and even around the vessels [43]. The metaphysis is associated with high vascularization of arterial blood, which supports tumor growth by providing nutrients and growth factors. High vascularization also leads to high oxygen levels in the metaphysis, while the diaphysis is associated with low oxygen levels and hypoxia. These properties make the metaphysis an attractive anatomical location for the cancer cells to home to and to grow.

The bone marrow vasculature has also been associated with tumor cell dormancy. Price et al. demonstrated that dormant and proliferating tumor cells localize in distinct areas of the bone marrow [41]. Dormant tumor cells were mainly found in the perisinusoidal vascular areas of the bone, which are rich in E-selectin and SDF-1. The authors showed that E-selectin is important for the cancer cells to enter the bone via the sinusoidal niche. In contrast, SDF-1 interactions were not involved in the dissemination process, but were essential for the disseminated cancer cells to remain in the skeleton. These findings suggest that the inhibition of vascular E-selectin and the SDF-1 receptor CXCR4 can prevent metastasis to the skeleton [41]. The role of bone marrow vasculature in tumor dormancy was also investigated by Ghajar et al. They demonstrated that proliferation of the primary tumor cells is independent of vascular proximity [84]. However, disseminated breast cancer cells were found on the microvasculature of different organs including the bone marrow, after metastasis. Additionally, endothelial cells of the bone marrow induced dormancy of cancer cells through the expression of the tumor suppressor thrombospondin-1 (TSP-1) [84]. Initiation of neovascular sprouting resulted in loss or reduction of the endothelial derived TSP-1 which facilitated the cancer cells to escape dormancy and enabled metastatic growth. These vascular subniches of neovascularization were found to be rich in TGF-β1, periostin, tenascin, versican, and fibronectin that further promote tumor growth [84].

### 6.2. The Effect of Hypoxia in Metastatic Bone Environment

The levels of oxygen supply vary between the different bone marrow compartments. Hypoxia (<5% oxygen) can be detected via HIFs, heterodimeric transcriptional regulators that are composed of one of three alpha subunits (HIF1α, HIF2α or HIF3α), and a beta subunit (HIF1β). Under normal oxygen supply, termed normoxia, the subunits HIF1α and HIF2α are hydroxylated and recognized by Von Hippel–Lindau (VHL) tumor suppressor protein which is part of E3 ubiquitin protein ligase. Ubiquitinylated HIF1α is degraded by the proteasome. Under hypoxia, HIF1α and HIF2α are not degraded anymore, and therefore they can bind to the HIF1β subunit [14]. HIF signaling has an important role in physiological bone formation. Decreased bone volume was detected after conditional loss of *HIF-1α* or *HIF-2α* in osteoblasts [85] while knockout of *VHL* resulted in increased levels of HIF-1α or HIF-2α leading to an increase in bone volume [86].

HIF-1 regulation directly activates transcription of genes involved in tumor metabolism and glycolysis, angiogenesis, tumor cell survival, and proliferation, as well as tumor invasiveness and metastasis [87]. It is well known that cancer cells have the ability to adapt to and survive at low oxygen levels. Consistently, overexpression of HIF1α is associated with poor prognosis, treatment resistance and failure, enhanced invasiveness and metastasis, and increased mortality in different types of cancer including breast cancer [88]. Hypoxia also induces angiogenesis by the upregulation of VEGF. Consequently, reduced angiogenesis and osteogenesis were observed with loss of HIF1α in long bones, whereas reversed effects were found with loss of VHL [86]. In addition, HIF1α promotes the secretion of MMP1 and MMP2. Increased abundance of VEGF and MMPs leads to (micro)vascular permeability which could promote intravasation and extravasation of tumor cells to the bone [89,90,91]. Indeed, HIF1α overexpression stimulates bone metastases of breast cancer cells [92], whereas knockdown of HIF1α showed a decrease of metastatic growth [93].

As discussed earlier, the CXCR4/CXCL12 axis promotes tumor cell homing to bone. Intriguingly, hypoxia stimulates CXCR4 expression in breast cancer, thereby promoting homing of metastatic breast cancer cells [94]. Similar findings were also made by Devignes et al., who demonstrated that HIF signaling increases the secretion of CXCL12 by osteoprogenitors into the bloodstream [42]. Upregulation of CXCL12 promoted breast cancer cell dissemination and growth in the skeleton. Interestingly, HIF-signaling in osteoprogenitor cells not only promoted metastasis in the bones, but also stimulated breast cancer cell dissemination to organs beyond the skeleton, for instance the lung [42].

Low oxygen tension has also been proposed to regulate DTC dormancy. In support of this hypothesis, Johnson and colleagues demonstrated that the prodormancy factor leukemia inhibitory factor (LIF) receptor (LIFR) was downregulated under hypoxic conditions. LIF is produced by the cells of the osteoblast lineage and by bone marrow stromal cells. Loss or downregulation of LIFR or its downstream signaling molecule STAT3 resulted in an exit of a dormancy state leading to an invasion and migration of breast cancer cells to the bone. Thus, these data suggest that patients with reduced LIFR expression more likely develop bone metastasis as compared with patients with normal LIFR expression [95].

## 7. The Role of Nerve Cells in Bone Metastases

Several factors, including traumatic emotional events, stress, and depression result in prolonged activation of the sympathetic nervous system [18]. Activation of the sympathetic nervous system has also been shown to be involved in breast cancer metastasis to bone. Campbell et al. demonstrated that chronic immobilization stress resulted in metastasis of breast cancer cells and development of osteolytic lesions [17]. In this study, the sympathetic nervous system was activated through stress and altered the bone marrow stroma. These neuronal effects in the stroma stimulated MDA-MD-231 breast cancer cells to colonize to the bone. Furthermore, β2AR stimulation induced RANKL production by osteoblasts and increased MDA-MD-231 breast cancer cell migration independent of the CXCL12-CXCR4-axis in vitro. Propanolol, a β-blocker, as well as RANK knockdown inhibited this effect in vivo, suggesting the involvement of osteoblast-β2AR and sympathetic activation in bone colonization and metastatic growth [17].

As already described in previous chapters, MMPs play an important role in bone remodeling and metastasis. Interestingly, neurotransmitter and neural-related factors of the nervous system have been shown to regulate MMP expression. Glial cell line-derived neurotrophic factor (GDNF) has been shown to upregulate MMP-9 in pancreatic cancer suggesting a supportive function in tumor invasion [96]. In addition, nerve growth factor (NGF) upregulated MMP-2 expression and increased invasiveness in pancreatic cancer cells in vitro [97]. High levels of NGF, tropomyosin receptor kinase A (TrkA), or p75 neurotrophin receptor (p75NTR) are also associated with lymph node metastasis of breast cancer [98]. GDNF also activates rearranged during transfection (Ret), which belongs to the receptor tyrosine kinase (RTK) family [99]. Usually Ret is associated with endocrine tumors, but some breast cancer types express higher levels of Ret which is accompanied with a decreased metastasis free and overall survival. Inhibition of Ret led to a decreased abundance of focal adhesion kinase (FAK) and inhibited tumor growth and metastasis in a breast cancer metastasis model in vivo [99].

Interestingly, neurotransmitters and neuropeptides are also regulators of angiogenesis, and thus the nervous system has an influence on the vasculature [100]. Stimulation of the β-ARs increased the production of IL-6 in MDA-MB-231 and MDA-MB-231BR breast cancer cell lines and VEGF production in MDA-MB-231BR cells [101]. In addition, neuropeptide Y was shown to regulate angiogenesis via VEGF and to promote breast cancer growth [102]. Additionally, different types of nerve fibers are often located in a spiral way around vessels controlling the blood flow in various bone compartments. Therefore, vascular permeability can be increased by neuropeptides leading to permissive conditions for cancer cell colonization [19,103].

In addition to β-ARs, the stress-induced neurotransmitter norepinephrine also stimulates IL-6 production. Stromal cells of the tumor microenvironment, such as macrophages, express ARs and can be activated by neurotransmitters of the nervous system [103]. Sloan et al. showed that stress-induced, as well as pharmacological activation of β-ARs increased the CD11b^+^F4/80^+^ macrophage infiltration into the breast cancer parenchyma and lead to a 30-fold increase of metastasis to lymph nodes and lung. This effect could again be reversed using the β-blocker propanolol [104].

Stromal cells such as endothelial cells, immune cells, and fibroblasts also express α-adrenergic receptors (α-AR). Activation of α2-AR promoted tumor progression and metastasis in 4T1 breast cancer cells. Importantly, this effect was driven by stromal cells since the 4T1 breast cancer cells do not express adrenergic receptors [105]. Gumireddy et al. investigated the inhibitory neurotransmitter γ-aminobutyric acid (GABA) and its receptor γ-aminobutyric acid (GABA_A_) receptor alpha3 (Gabra3) that are in physiological conditions only expressed in the brain. Interestingly, Gabra3 is also expressed in breast cancer, but not in healthy epithelial breast tissue and is associated with poorer survival. Functionally, overexpression of Gabra 3 breast cancer cell lines MCF7 and MDA-MB-436 stimulated metastasis through AKT signaling [106]. These studies highlight the impact of the nervous system on breast cancer growth and metastasis by influencing the stroma, as well as the vasculature.

## 8. The Bone Marrow Microenvironment as a Target to Treat Breast Cancer Bone Metastases

Osteoclastic bone resorption is the hallmark of breast cancer bone metastasis and successful treatments have been developed to block the osteoclast–cancer cell interaction. These include, for example, bisphosphonates and the RANKL inhibitor denosumab, the current standard of care. These agents inhibit osteoclast activity and are capable of slowing down the tumor-induced osteolysis [107,108]. However, once the osteolytic lesions have developed, the disease remains incurable and treatment is restricted to palliative care. In addition, studies have focused on identifying alternative therapeutic approaches to block the vicious cycle by targeting osteoclasts. These include for example targeting cathepsin K (odanacatib), c-src (dasatinib, saracatinib), activin A (sotatercept), anti-avb3 integrin antibodies (etaracizumab), or mTOR (everolimus) [109,110,111] reviewed in [112].

Given the recently recognized role of osteoblasts in metastatic bone disease, osteoblasts are emerging as cellular targets to reduce disease progression and heal the osteolytic lesions. Recently, neutralizing antibodies against sclerostin have been developed as bone anabolic drugs to treat severe osteoporosis [113,114]. The Wnt antagonist sclerostin is secreted by osteocytes in the bone marrow and inhibits new bone formation by binding to low-density lipoprotein receptor 5 (LRP-5) on osteoblasts. Promising preclinical data have demonstrated a reduced metastatic breast cancer burden in the bones of female immune-compromised SCID mice, in addition to a prolonged survival upon treatment with anti-sclerostin antibody [115]. In addition, anti-sclerostin antibody treatment prevented breast cancer-induced bone loss by enhancing osteoblast function and simultaneously inhibiting bone resorption. Interestingly, sclerostin-mediated Wnt signaling is also involved in the differentiation and metabolism of adipocytes [116,117]. Consistently, anti-sclerostin antibody also reduces the volume of bone marrow adipose tissue [118,119], suggesting that the antitumor effect of sclerostin antibody in models of bone metastasis could also be mediated, at least in part, by the bone marrow adipocytes. Indeed, targeting adipocytes in the bone marrow in combination therapies could also emerge as a promising strategy to treat metastatic bone disease.

Osteocytes are also increasingly appreciated as novel, therapeutic targets for breast cancer bone metastasis. Osteocytes express connexin proteins (Cx), that are able to form hemichannels to allow the exchange of molecules between cells and their environment. Studies by Zhou et al. reported a role of osteocytic Cx43 hemichannels in suppressing breast cancer growth and bone metastasis [120]. Using the transwell and wound-healing migration assay the authors were able to show that conditioned medium from MLO-Y4 osteocytes that were treated with the bisphosphonate alendronate, dose dependently decreased the migration of MDA-MB-231 breast cancer cells in vitro. Using a hemichannel-blocking antibody the reduced breast cancer cell migration was attributed to the hemichannel-opening function of the bisphosphonate [120]. Interestingly, these results were not observed using the osteoblastic MLO-A5 cell line. Consistent with the reduced migration upon incubation in conditioned medium from MLO-Y4 cells treated with alendronate, a decreased colony formation of MDA-MB-231 cells was observed. Importantly, similar results were reported using the more potent bisphosphonate zoledronate, which is commonly used in the treatment of cancer induced bone disease [121,122,123,124]. Consistent with these in vitro findings, mice with impaired Cx43 gap junctions showed increased tumor burden in the long bones as compared with wild type mice. This suggests a protective role of Cx43 in osteocytes against breast cancer growth in vivo. In addition, there was a decrease in tumor growth-suppressing function of bisphosphonates in Cx43 knockout mice, supporting the notion that osteocytes mediate the tumor suppressive function of bisphosphonates in vivo via Cx43 hemichannels [120]. In addition, osteocytes might mediate the therapeutic effect of denosumab, an anti-osteolytic drug that has been approved to prevent skeletal-related events in patients with breast and prostate tumors, as well as bone metastases. Denosumab is a fully human monoclonal antibody against RANKL, one of the key drivers of the vicious cycle. Given that osteocytes are a major source of RANKL it is likely that treatment effects could be partially mediated by osteocytes.

Propranolol, a β-AR antagonist, is commonly used in the treatment of hypertension. Recent studies have shown that β-AR antagonists reduce breast cancer metastasis in vivo [17,104]. Additionally, a combination therapy of propranolol with metformin, a drug used in diabetes mellitus type 2, decreased migration, invasion, and proliferation of five breast cancer cell lines in vitro [125]. A clinical study conducted recently demonstrated that propranolol reduces Ki-67 and Bcl-2 markers in a stage III breast cancer patient. This was achieved through cell cycle disruption increasing the sub-G1 population. Additionally, propranolol stabilized p53 levels, and thus increased its phosphorylation resulting in proapoptotic signaling [126]. However, conflicting results were obtained in another clinical study which could not demonstrate an improved survival in breast cancer patients treated with propranolol [127]. By the end of 2016, propranolol was approved in the Orphan Drug Designation by European Medicines Agency (EMA) in soft tissue sarcoma. Whether the drug can be used to treat breast cancer bone metastasis remains to be investigated in the future.

In addition to directly targeting cells of the bone microenvironment, different drugs and agents have been tested to target hypoxic signaling. These approaches include targeting HIF1α, hypoxia-activated prodrugs, and gene therapy. However, since hypoxia and HIF1α are involved in many signaling pathways and target up to 60 genes [87], hypoxia inhibition alone might be an insufficient treatment option for bone metastasis [128].

## 9. Conclusions

The studies described in this review clearly demonstrate that various bone marrow niches including the endosteal niche (osteoblasts, osteoclasts, and adipocytes) and the vascular niche participate in the regulation of breast cancer dissemination, dormancy, and growth in bone (Figure 1, Table 1). In the past independent niches were considered to be responsible for certain cancer cell properties, however, recent evidence suggest that there is a significant overlap between different niches [43], and thus cancer cells are affected by a complex mixture of different cell types in the bone marrow microenvironment. In addition, although bone has been considered as a fertile soil for tumor cell invasion and metastasis for decades, the underlying cause for this “attractiveness” is still not fully understood. While the crucial function of osteoclasts in bone metastases activation and development is well established, further efforts are needed to elucidate the contribution of other cells of the complex bone microenvironment to breast cancer bone metastasis. Especially the role of adipocytes and nerve cells remains poorly understood. Increasing our knowledge on how these cells interact with disseminated tumor cells could help to identify novel therapeutic strategies to interfere with the earliest steps of bone metastasis and consequently prevent disease establishment.

## Figures and Tables

**Table 1:** Summary of key publications highlighting the contribution of the bone microenvironment to the development and progression of bone metastasis.

Cell/Molecule	Key Findings	Reference
**Bone Cells**		
Osteoclasts	Osteoclasts are not involved in tumor cell homing, but drive osteolysis	[34,35]
Osteoblasts	Interaction between osteoblasts and breast cancer cells via CXCL12/CXCR4 is important for tumor cell homing	[41]
	Breast cancer cells modify osteoblast migration	[46]
	Co-injection of breast cancer cells and osteoblasts promotes tumor growth	[47]
	IL-6, IL-8, MCP-1, MIP-2 and VEGF are increased in osteoblasts in the presence of breast cancer cells	[44,48]
	OPN^high^ and aSMA^low^ osteoblasts decrease cancer cell proliferation and may regulate dormancy	[35]
	Cancer cells alter osteoblast arrangement and collagen organization	[54,55,56]
Osteocytes	Osteocytes secrete RANKL, MMPs, TNFα, sclerostin which influence cancer cell proliferation and migration	[24,62]
	Osteocyte conditioned medium increased proliferation of human prostate and breast cancer cells	[63]
**Vasculature**		
Type H/type L capillaries	Expression of CD31/endomucin distinguishes vessels in metaphysis and diaphysis	[13]
Endothelial cells	Breast cancer cells localize in metaphysis and around the vasculature	[43,44]
	Endothelial cells regulate cancer cell dormancy via thrombospondin-1	[84]
**Hypoxia**		
HIF signaling	HIF1α overexpression stimulates bone metastasis, HIF1α knockdown shows reverse effects	[92,93]
	HIF signaling stimulates cancer cell dissemination to bone via CXCR4/CXCL12	[42,95]
LIFR/STAT3	Loss of LIFR/STAT3 regulates dormancy escape of breast cancer cells	[95]
**Nerve Cells**		
β2AR	Stimulates RANKL production and metastasis	[17]
GDNF, NGF	Increase invasiveness of pancreatic cancer cells via MMP2 and MMP9	[97,98]
Neurotransmittors, Neuropeptides, βAR,	Regulation of VEGF and angiogenesis	[19,101,102,103]
α2-AR	Stromal cells expressing α2-AR promote breast cancer cell metastasis	[105]
Gabra3	Overexpression stimulates metastasis via AKT signaling	[106]
**Adipocytes**		
Lipids	Lipids act as energy source for cancer cells, affect their metabolism and increase their invasiveness	[67,72,73,74]
Adipokines	Adiponectin has anti-tumoral effects via mTOR and NF-_K_B signaling	[75]
	Leptin promotes metastasis via regulation of VEGF	[80]
